# Differences Changes in Cerebellar Functional Connectivity Between Mild Cognitive Impairment and Alzheimer's Disease: A Seed-Based Approach

**DOI:** 10.3389/fneur.2021.645171

**Published:** 2021-06-17

**Authors:** Fanyu Tang, Donglin Zhu, Wenying Ma, Qun Yao, Qian Li, Jingping Shi

**Affiliations:** ^1^Department of Neurology, Affiliated Brain Hospital of Nanjing Medical University, Nanjing, China; ^2^Department of Neurology, Affiliated to Nanjing Medical University, Nanjing, China; ^3^Nanjing Medical University, Nanjing, China

**Keywords:** cerebellum, Azheimer's disease, mild cognitive impairment, functional connectivity, resting-state functional MRI

## Abstract

**Background:** Recent studies have discovered that functional connections are impaired among patients with Alzheimer's disease (AD), even at the preclinical stage. The cerebellum has been implicated as playing a role in cognitive processes. However, functional connectivity (FC) among cognitive sub-regions of the cerebellum in patients with AD and mild cognitive impairment (MCI) remains to be further elucidated.

**Objective:** Our study aims to investigate the FC changes of the cerebellum among patients with AD and MCI, compared to healthy controls (HC). Additionally, we explored the role of cerebellum FC changes in the cognitive performance of all subjects.

**Materials:** Resting-state functional magnetic resonance imaging (rs-fMRI) data from three different groups (28 AD patients, 26 MCI patients, and 30 HC) was collected. We defined cerebellar crus II and lobule IX as seed regions to assess the intragroup differences of cortico-cerebellar connectivity. Bias correlational analysis was performed to investigate the relationship between changes in FC and neuropsychological performance.

**Results:** Compared to HC, AD patients had decreased FC within the caudate, limbic lobe, medial frontal gyrus (MFG), middle temporal gyrus, superior frontal gyrus, parietal lobe/precuneus, inferior temporal gyrus, and posterior cingulate gyrus. Interestingly, MCI patients demonstrated increased FC within inferior parietal lobe, and MFG, while they had decreased FC in the thalamus, inferior frontal gyrus, and superior frontal gyrus. Further analysis indicated that FC changes between the left crus II and the right thalamus, as well as between left lobule IX and the right parietal lobe, were both associated with cognitive decline in AD. Disrupted FC between left crus II and right thalamus, as well as between left lobule IX and right parietal lobe, was associated with attention deficit among subjects with MCI.

**Conclusion:** These findings indicate that cortico-cerebellar FC in MCI and AD patients was significantly disrupted with different distributions, particularly in the default mode networks (DMN) and fronto-parietal networks (FPN) region. Increased activity within the fronto-parietal areas of MCI patients indicated a possible compensatory role for the cerebellum in cognitive impairment. Therefore, alterations in the cortico-cerebellar FC represent a novel approach for early diagnosis and a potential therapeutic target for early intervention.

## Introduction

Alzheimer's disease (AD), the most common neurodegenerative dementia, is characterized by a progressive deterioration of cognitive functions, as well as changes in behavior and personality (1, 2). Amnesic mild cognitive impairment (aMCI) has been recognized as a transition stage between normal cognitive function and AD-type dementia, which has a chance of progression to AD up to 25% per year (3). Resting-state functional magnetic resonance imaging (fMRI) is reflecting the synchronization of functional activity between distant brain regions by observing the brain low frequency fluctuations in the blood-oxygen level-dependent (BOLD) signals, which is widely used in diagnosis and predict the disease progression of AD (4–6). The application of resting-state fMRI techniques has revealed imaging features of AD with regards to brain structure (7, 8). Studies have shown that functional connections and brain networks are impaired as early as the aMCI stage (5). In the preclinical stage of AD, the hippocampus, visual cortex and frontal lobe have been decoupled, and the enhanced connections between the middle cingulate gyrus (MCC), the precuneus gyrus (PCU), the posterior cingulate gyrus (PCC), and the cerebellum are the internal mechanisms of ad functional compensation. After entering MCI stage, PCU can no longer compensate for the decompensation of AD susceptible areas such as hippocampus, but the connection between cerebellum, MCC and PCC is enhanced, which can be maintained until dementia stage (9, 10). AD progression can be delayed via early diagnosis and intervention. Resting state fMRI can be more sensitive to explore the brain network changes of early AD (11, 12). Previous studies have focused on the cerebral cortex, and less research has been focused on the role of the cerebellum in cognitive regulation of the AD spectrum (13, 14).

Recent studies have implicated the role of cerebellum in cognitive processes (15–19). Cerebellar cognitive affective syndrome (CCAS) is characterized by executive dysfunction, spatial cognitive impairment, language deficits, and personality changes (20–22). The human cerebellar cortex is a complicated structure, as the surface of it is more tightly folded than the cerebral cortex, and has almost 80% of the surface area of the neocortex, and the nerve fiber connections to the brain's cognitive network is extensive (23), indicating that the cerebellum plays an important role in the evolution of behavior and cognition. Furthermore, the cerebellar lobular volumes, as well as the cortico-cerebellar FC, was found to decrease with age, leading to cognitive decline among the healthy elderly people (24, 25). Previous studies have suggested that the cerebellum is a survivor of preclinical AD stage process and remains virtually unaffected (26, 27). However, recent studies have found that as the disease progresses, the structure, as well as the function of the cerebellum also changes (28).

With the progression of AD, the cerebellar gray matter volume changes in a continuum with posterior-to-anterior cerebellar lobe development. The vermis and paravermian lobes of anterior lobe (I-V) and posterior lobe (VI) were mainly involved in aMCI, and the hemispheric part of posterior lobe (VI lobule) and Crus I were involved in AD. GM atrophy of Crus I will cause functional damage, which becomes more obvious with the increase of disease severity (29). Another study shows that the FC between the dentate nucleus (DN) and lateral temporal regions was increased in AD patients compared to controls, when using cerebellar DN as a region of interest, which suggests that FC changes within specific cerebellar-cortical functional modules is involved in cognitive impairment among AD patients (30). The DN is involved in planning and execution of random movements, as well as higher cognitive and sensory processing. It is also a key area involved in integration and regulating cerebral-cerebellar networks. The dorsolateral prefrontal lobe of the cerebral cortex is involved in working memory, decision-making, time processing, and other cognitive functions (31). After an injection of a trans-neuronal retrograde tracer to the dorsolateral prefrontal lobe, a small number of Purkinje cells were labeled in the lateral region of Crus II (32) (along with Crus I, a hemispheric extension of lobule VIIa), as well as in the normal portions of lobule X and lobule VII. This suggests the existence of a “cognitive” loop between dorsolateral prefrontal lobe and specific cerebellar cortical areas (33).

An increasing number of imaging studies have confirmed that cerebellar Crus II and lobular IX are associated with cognitive networks (32, 34, 35), particularly with the default mode network (DMN) and fronto-parietal network (FPN) (36–38). The anterior cerebellum and lobule VIII are associated with movement, whereas the posterior cerebellum (i.e., lobule VI-Crus I, lobule Crus II-VIIB, and lobule IX) are critical for cognitive representation (39–41). Lobule VI, VIIB, and Crus I are specifically involved in executive functions, including working memory, planning, organizing, and strategy formation, all of which are important for creative divergent thinking (42–45). Visual divergent thinking is an approach to a situation or concept that focuses on exploring as many aspects of the visual concept as possible, and it is a primary component of fields such as photography, drawing, architecture and sculpture, which is significantly associated with activity in the left lobule VI, VIIB, Crus I, and Crus II, and is associated with executive function (46). Previous studies have established that working memory task processing and front-oparietal network connectivity simultaneously engaged lobule VI/Crus I, Crus II/lobule VIIB, and lobule IX with the DMN (32, 44, 47). Bai et al. used a resting-state fMRI to explore spontaneous activation of the cerebellum and found significant differences in lobule IX and Crus II in the posterior cerebellum of aMCI patients compared to controls (36). Therefore, bilateral Crus II and lobule IX were chosen herein as regions of interest to study FC characteristics of the cerebellum in patients with aMCI and AD.

However, the characteristics and differences of cerebellar FC in MCI and AD patients remains unknown. This present study was conducted to analyze alterations in cerebellar-cortical FC and whether these alterations were associated with clinical cognitive impairment in MCI and AD patients.

## Materials and Methods

### Participants

All study subjects were recruited from the Nanjing Brain Hospital between June 2018 to October 2020. All subjects were right-handed, and included 27 AD subjects (11 males and 16 females), 25 MCI subjects (9 males and 16 females), and 13 healthy controls were not different statistically in terms of age and sex (7 males and 6 females). The diagnosis of AD and MCI was carried out according to the National Institute on Aging and the Alzheimer's Association working group (NIA-A) in 2011 (3, 48). All patients underwent clinical and neuropsychological assessment, MRI scans, and cerebrospinal fluid (CSF) analysis. The inclusion criteria for health controls (HC) included (1) no current cognitive issues, (2) no neurological or psychiatric diseases, and (3) a clinical dementia rating (CDR) score of 0 (49). Exclusion criteria for both groups included (1) other known causes of dementia (i.e., frontotemporal dementia, dementia with Lewy bodies, vascular dementia, severe depression, cerebrovascular disease, tumors, poisoning, metabolic diseases, and infections), and (2) contraindications to undergoing an MRI, such as claustrophobia or pacemaker implantation.

All participants provided a written informed consent and the study was granted approval by the Medical Research Ethical Committee of Nanjing Brain Hospital in Nanjing, China.

### Clinical and Neuropsychological Assessments

All participants underwent comprehensive and standard neuropsychological assessments in order to evaluate their cognitive function. Global cognition was evaluated using the mini-mental state examination (MMSE) (50), the Montreal cognitive assessment (MoCA) (51), and the CDR (52). Episodic memory was assessed using the auditory verbal learning test (AVLT) (53). Visuospatial abilities were evaluated with the clock-drawing test (CDT) (54). Language function was determined using the Boston naming test (BNT) and the verbal fluency test (VFT-animals). Executive function was assessed using part A and B of the trail making test (TMT), as well as the symbol digit modalities test (SDMT). Verbal working memory was determined using the digit span test (DST) (55). The emotional condition of the subjects were determined using the Hamilton Depression (HAMD) (56). These scales were validated by senior neuropsychologists and evaluated by experienced clinicians.

### Cerebrospinal Fluid Biomarkers

CSF Aβ1–42, t-tau, and p-tau were measured using INNOBIA AlzBio3 immunoassay kit-based reagents (Innotest, Fujirebio, Ghent, Belgium). Notably, not all participants had CSF sample data since lumbar puncture is an invasive operation. In this study, 25AD subjects and 27 MCI subjects had CSF sample data available.

#### Magnetic Resonance Imaging Data Acquisition

Magnetic resonance imaging (MRI) was acquired utilizing a Siemens 3.0 T singer scanner (Siemens, Verio, Germany) with an 8-channel radio frequency coil at the Affiliated Brain Hospital of Nanjing Medical University. Participants were asked to remain as still as possible, close their eyes, remain awake, and to not think of anything. T1WI was acquired through application of a three-dimensional magnetization prepared rapid gradient echo (3D-MPRAGE). The parameters included time repetition (TR) = 1,900 ms, echo time (TE) = 2.48 ms, inversion time (TI) = 900 ms, number of slices = 176, thickness = 1.0 mm, gap = 0.5 mm, matrix = 256 × 256, flip angle (FA) = 9?, field of view (FOV) = 256 × 256 mm, and voxel size = 1 × 1 × 1 mm^3^. Resting-state fMRI acquisition was applied using single echo planar imaging (EPI). The gradient echo-echo planar imaging (GRE-EPI) sequence included 240 time points. TE = 30 ms; TR = 2,000 ms; number of slices = 36, FOV = 220 × 220 mm^2^; matrix = 64 × 64; FA = 90°; thickness = 4.0 mm, gap = 0 mm. The imaging for each subject took ~ 14 min.

#### Data Preprocessing

The fMRI data were processed using the Data Processing and Analysis for Brain Imaging (DPABI, http://www.rest.restfmri.net) (57). The first 10 volumes of the rest session were discarded for each subject. The remaining images were corrected utilizing slice timing and motion (head motion ≤ 3 mm, head motion angle ≤ 3°). Next, resting-state fMRI images were co-registered to high-resolution 3D-T1 structural images. Normalization of 3D-T1 structural MRI images to Montreal Neurological Institute (MNI) space was undertaken via non-linear warping based on Diffeomorphic Anatomical Registration Through Exponentiated Lie Algebra (DARTEL). After spatial normalization to T1 space, all images were resampled into 3 × 3 × 3 mm^3^ voxels and spatially smoothed using a Gaussian filter of 6 mm full-width at half-maximum (FWHM). Data was then temporally band-pass-filtered (0.01–0.08 Hz) in order to eliminate low-frequency drifts and physiological high-frequency noise. Furthermore, to reduce the confounding artifacts of resting head movements and physiological noise (respiration and cardiac fluctuations), nuisance covariates were regressed out, including the Friston 24-motion parameter model, global mean, white matter, and cerebrospinal fluid signals.

#### Functional Connectivity Analysis

**Figure 1:**
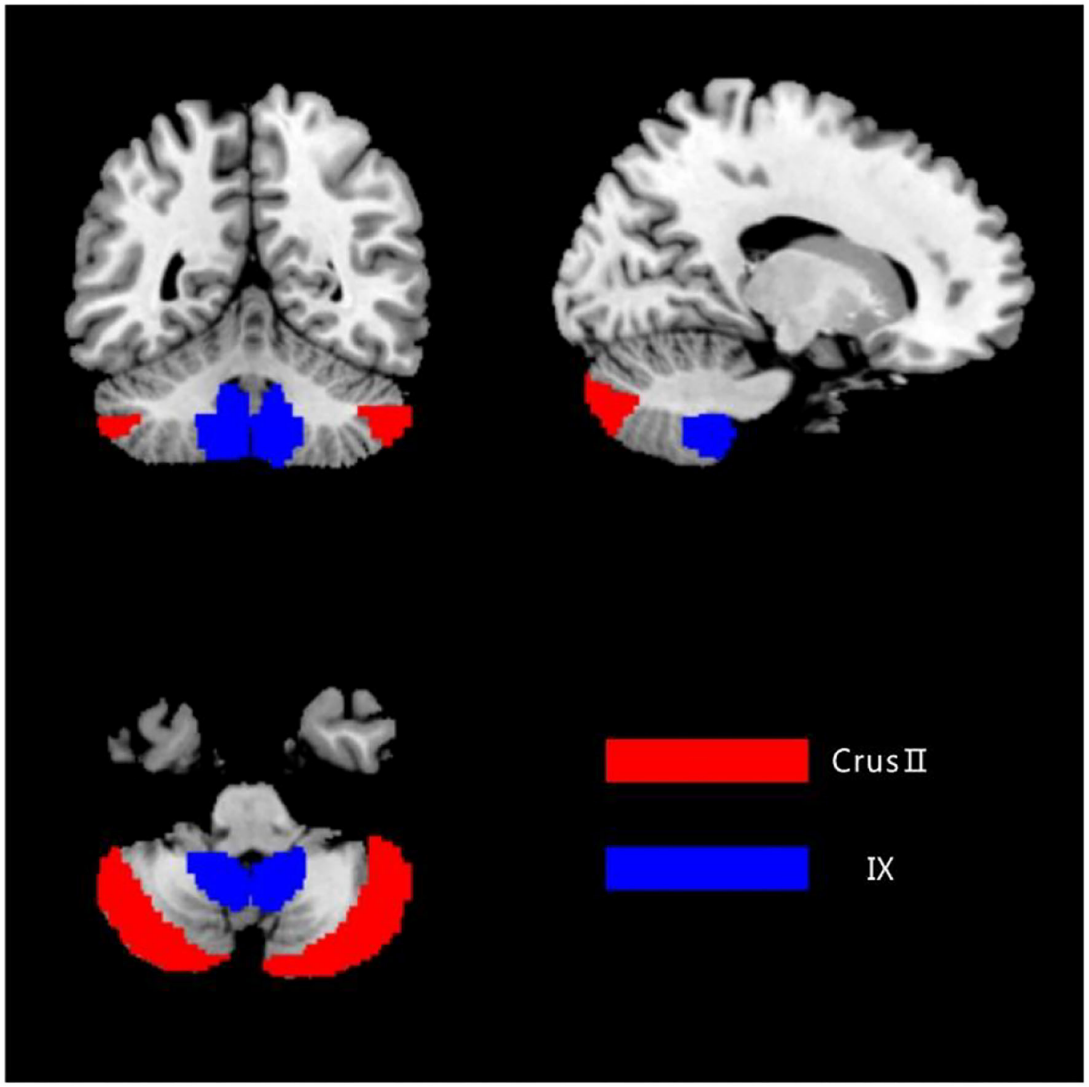
The ROIs of the cerebellum. The image was transformed into the space of the SUIT atlas and was overlapped by the seeds. Red color represents the Crus II, and blue color represents the posterior lobule IX.

The bilateral cerebellar Crus II and lobule IX were extracted as regions of interest (ROI) utilizing the DPABI software package template (anatomical automatic labeling, AAL) in order to localize the two ROIs, respectively (Figure 1).

FC analysis was performed between each seed region, as well as the whole brain, in a voxel-wise manner using the DPABI software. The voxels of each seed region of every subject were extracted and averaged in order to obtain the reference time series of seed points. Then, we calculated the correlation coefficient between the reference time series and the time series involving all other brain voxels. The correlation coefficients were transformed into *z*-values using the Fisher *r*-to-*z* transformation, leading to an improvement in normality.

#### Statistical Analysis

All data was tested for normality and variance congruence. Normally distributed data was expressed as mean ± standard deviation. The ANOVA and chi-square test were performed to compare the demographic and neurocognitive data among the groups (AD, MCI, and HC). Bonferroni correction was used for *post-hoc* comparisons. Statistical analyses were performed using IBM SPSS 25.0 software (SPSS Inc., Chicago, Illinois, USA). A two-sided *P* < 0.05 represented statistical significance.

In order to determine the differences of whole-brain resting-state FC of each cerebellar seed region, we conducted statistical analyses across the three groups was conducted utilizing ANOVA, with age, sex, years of education, and gray matter volume used as covariates. Gray matter volume sequence was extracted by REST Toolkit (http://www.restfmri.net). The multiple comparisons of ANOVA results were corrected using AlphaSim with a significance threshold of *P* < 0.05 (cluster size > 100 voxels, and voxel-level *P* < 0.05; determined by a Monte Carlo simulation). *Post-hoc* two-sample *t* tests were corrected by Bonferroni. *P* < 0.05 was statistically significant.

Then, partial bias correlation analysis was utilized to explore the relationship between resting-state FC among different brain regions with the clinical neuropsychological score, using gender, age, and education level as covariates (*P* < 0.05, Bonferroni-corrected).

## Results

### Demographic and Clinical Characteristics

The demographic and clinical characteristics of each participant are indicated in Table 1. There were no significant differences with regards to age, gender, or education among the three groups. In contrast, there were significant differences for each cognitive domain. Overall cognitive levels, episodic memory ability, executive ability, verbal ability, and visuospatial function were significantly lower in the AD group compared to both the MCI and HC group. There was also a significant decrease in the above cognitive domains in the MCI group compared to the HC group (*p* < 0.001).

**Table 1:** Demographic and clinical characteristics of the participants.

	**AD**	**MCI**	**Control**	***F***	***P***
	**(*n* = 27)**	**(*n* = 25)**	**(*n* = 20)**		
Age (years)	64.81 ± 8.24	66.92 ± 8.96	62.60 ± 6.95	1.557	0.251
Gender (male/female)^d^	11/16	9/16	11/9	0.85	0.432
Education (years)	7.59 ± 5.54	10.20 ± 3.18	8.65 ± 4.62	2.349	0.097
CSF t-tau (pg./ml)^e^	513.46 ± 144.68	473.33 ± 170.52	–	0.917	0.363
CSF p-tau (pg./ml)^e^	115.05 ± 67.46	110.17 ± 58.85	–	0.277	0.783
CSF β_1−42_ (pg./ml)^e^	542.96 ± 241.18	557.51 ± 311.76	–	0.189	0.851
MMSE	12.48 ± 5.23	22.44 ± 2.86	27.15 ± 1.35	99.487	<0.001^a,b,c^
MOCA	7.19 ± 4.28	16.04 ± 3.53	25.23 ± 1.36	159.92	<0.001^a,b,c^
HAMD	5.96 ± 4.49	3.80 ± 2.94	2.85 ± 1.46	5.895	0.004
AVLT-A (memory)	0.41 ± 0.97	0.36 ± 0.91	4.62 ± 1.12	125.463	<0.001^a,b^
AVLT-B (memory)	10.67 ± 6.96	16.80 ± 2.94	19.54 ± 2.63	25.615	<0.001^a,c^
CDT (visual spatial memory)	9.52 ± 9.74	22.04 ± 7.15	25.00 ± 3.14	33.09	<0.001^a,c^
SDMT (attention)	4.41 ± 6.93	19.56 ± 11.45	29.15 ± 5.97	58.941	<0.001^a,b,*,c^
DST (attention)	4.26 ± 2.35	7.32 ± 1.87	10.08 ± 2.25	47.184	<0.001^a,b,c^
BNT (verbal naming ability)	13.52 ± 4.72	19.76 ± 4.74	22.69 ± 2.84	32.145	<0.001^a,b,c^
VFT (verbal fluency)	7.19 ± 3.32	11.16 ± 3.97	10.18 ± 4.47	30.78	<0.001^a,b,c^
TMT-A (Attention)	301.41 ± 137.86	123.04 ± 34.66	65.35 ± 11.53	48.54	<0.001^a,b,*,c^
TMT-B (Executive)	466.85 ± 115.14	307.56 ± 80.32	118.90 ± 18.11	95.03	<0.001^a,b,c^

*MMSE, Mini-Mental State Examination; MoCA, Montreal Cognitive Assessment; HAMD, Hamilton Depression Scale; AVLT, Auditory Verbal Learning Test; CDT, Clock Drawing Test; SDMT, Symbol Digit Modalities Test; DST, Digit Span Test; BNT, Boston Naming Test; VFT, Verbal Fluency Task; TMT, Trail-Making Test*.

a *Post-hoc analysis showed significant group differences between HC and MCI*.

b *Post-hoc analysis showed significant group differences between HC and AD*.

c *Post-hoc analysis showed significant group differences between MCI and AD*.

d *Values were mean ± standard deviation. Comparisons using Chi-square test*.

e *Values were mean ± standard deviation (SD). Comparisons using Student's t test*.

* *Means p < 0.05*.

### Functional Connectivity

#### Functional Connectivity Changes of the Left Crus II

**Figure 2:**
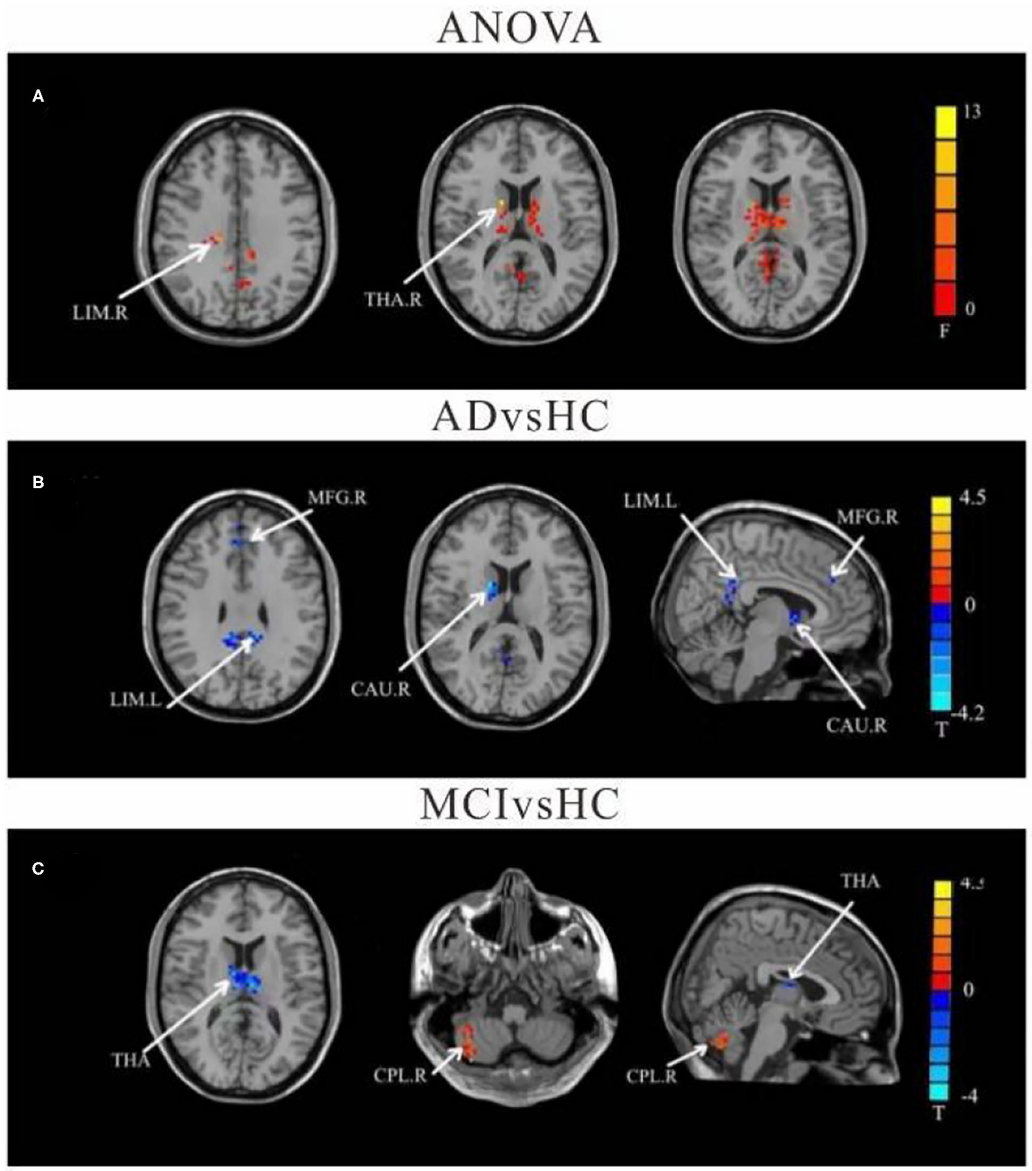
Differences of FC values in the cerebellum left crus II. **(A)** Brain regions showing significant differences in FC of the left cerebellum crus II based on analysis of variance (ANOVA) analysis between HC, AD, and aMCI, *p* < 0.05, the cluster size > 100 voxels). **(B,C)** Results of *post-hoc* two-sample *T*-tests in voxel-wise analysis (Bonferroni corrected, cluster size ≥ 30 voxels, *p* < 0.05). AD, Alzheimer's disease; aMCI, amnestic mild cognitive impairment; HC, healthy controls; LIM, limbic lobe; THA, Thalamus; MFG, Middle Frontal Gyrus; CAU, Caudate; CPL, cerebellum posterior lobe; L, left; R, right.

Using left crus II as the ROI, whole-brain FC analysis revealed that FC values of the right caudate (CAU), left limbic lobe (LIM), and right medial frontal gyrus (MFG) were reduced in AD patients compared to HC. Furthermore, FC with left thalamus (THAL) was decreased in the MCI group, but increased in the right cerebellum posterior lobe (CPL) compared to the HC group. There were no significant differences in left-sided crus II and whole brain FC between the AD and MCI groups (Table 2 and Figure 2).

**Table 2:** Regions showing resting state functional connectivity (rs-FC) changes in cerebellum left crus II.

**Brain region (aal)**	**Cluster size**	**MNI coordinates**			***T***
		**X**	**Y**	**Z**	
**ANOVA**					
R-Thalamus	147	15	3	15	14.74
R-Limbic lobe	132	15	−24	33	10.57
**AD < Controls**					
R-Caudate	43	15	3	15	−4.65
L-Limbic lobe	94	−9	−36	30	−4.02
R-Medial frontal gyrus	37	3	45	42	−3.44
**aMCI < Controls**					
L-Thalamus	44	−6	−15	12	−4.52
R-Cerebellum posterior lobe (VIII)	31	36	−78	−54	3.41
R-Cerebellum posterior lobe (VIII)	32	9	−69	36	3.75

*MNI, Montreal Neurological Institute; Cluster size > 100 voxels in ANOVA analysis. Significance set at p < 0.05 corrected by AlphaSim*.

*Cluster size > 30 voxels in two-sample T-test*.

*Significance set at p < 0.05 corrected by Bonferroni*.

#### Functional Connectivity Changes of the Right Crus II

**Figure 3:**
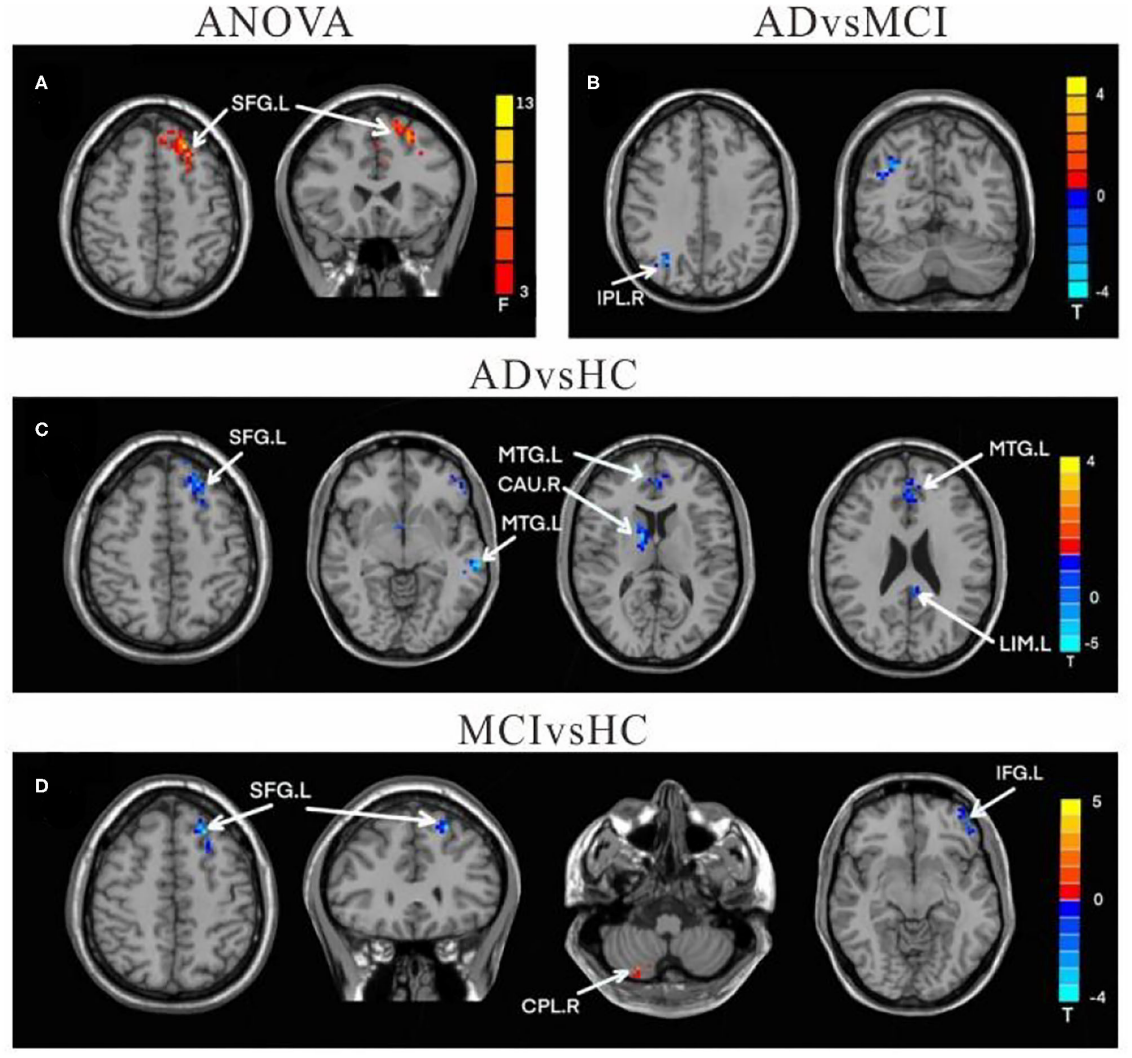
Differences of FC values in the right cerebellum Crus II. **(A)** Brain regions showing significant differences in FC of the right cerebellum Crus II based on analysis of variance (ANOVA) analysis between HC, AD, and aMCI patients, *p* < 0.05, the cluster size > 100 voxels). **(B–D)** The results of *post-hoc* two-sample *T*-tests in voxel-wise analysis (Bonferroni corrected, cluster size ≥ 30 voxels, *p* < 0.05). AD, Alzheimer's disease; aMCI, amnestic mild cognitive impairment; HC, healthy controls; SFG, superior frontal gyrus; IPL, inferior parietal lobe; MFG, Middle Frontal Gyrus; CAU, Caudate; LIM, limbic lobe; CPL, cerebellum posterior lobe; IFG, inferior frontal lobe; L, left; R, right.

Compared to HC, the FC values in AD between right crus II and left middle temporal gyrus (MTG), left MFG, right CAU, left superior frontal gyrus (SFG), and left LIM were significantly decreased. Among individuals with MCI, decreased FC was indicated between the right crus II with left inferior frontal gyrus (IFG) and left superior frontal gyrus (SFG), while the right CPL FC were increased among individuals with MCI. The FC changes in right crus II and inferior parietal lobe (IPL) was decreased in AD and MCI patients (Table 3 and Figure 3).

**Table 3:** Regions showing resting state functional connectivity (rs-FC) changes in cerebellum right crus II.

**Brain region (aal)**	**Cluster size**	**MNI coordinates**			***T***
		**X**	**Y**	**Z**	
**ANOVA**					
L-Superior frontal gyrus	300	−21	30	51	12.8
**AD < Controls**					
L-Middle temporal gyrus	39	−63	−30	−6	−4.61
L-Middle frontal gyrus	38	−45	51	−9	−3.39
L-Medial frontal gyrus	81	0	57	27	−3.73
L-Middle frontal gyrus	39	−42	21	33	−3.68
R-Caudate	62	12	0	12	−4.18
L-Superior frontal gyrus	76	−24	21	45	−4.07
L-Limbic lobe	31	−12	−39	30	−3.75
**aMCI < Controls**					
R-Cerebellum posterior lobe (VIII)	33	39	−60	−54	3.26
R-Cerebellum posterior lobe (VIII)	31	24	−75	−51	3.17
L-Inferior frontal gyrus	31	−48	45	−9	−3.29
L-Superior frontal gyrus	53	−21	30	51	−4.03
**AD < aMCI**					
R-Inferior parietal lobule	30	30	−66	36	−3.59

*MNI, Montreal Neurological Institute; Cluster size > 100 voxels in ANOVA analysis. Significance set at p < 0.05 corrected by AlphaSim. Cluster size > 30 voxels in two-sample T-test. Significance set at p < 0.05 corrected by Bonferroni*.

#### Functional Connectivity Changes of the Left Lobule IX

**Figure 4:**
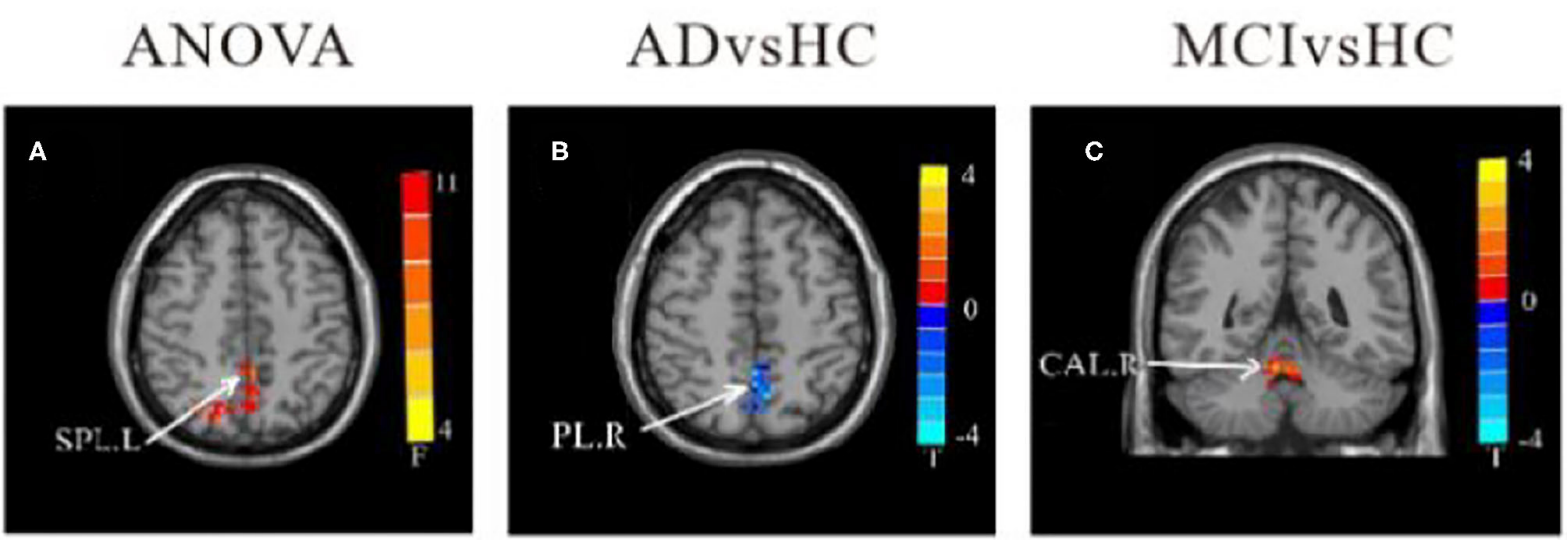
Differences of FC values in the cerebellum left lobule IX. **(A)** Brain regions showing significant differences in FC of the cerebellum left lobule IX based on analysis of variance (ANOVA) analysis between HC, AD, and aMCI (*p* < 0.05; the cluster size > 100 voxels). **(B,C)** The results of *post-hoc* two-sample *T*-tests in voxel-wise analysis (Bonferroni corrected, cluster size ≥ 30 voxels, *p* < 0.05). AD, Alzheimer's disease; aMCI, amnestic mild cognitive impairment; HC, healthy controls; SPL, superior parietal lobe; PL, parietal lobe; CAL, cerebellum Anterior lobe; L, left; R, right.

In comparison to HC, the FC value between cerebellum lobule IX and right parietal lobe/precuneus (PCU) was remarkably reduced in the AD group. In contrast, FC values in left lobule IX and right cerebellum anterior lobe (CAL) were significantly increased in MCI patients compared to HC. There were no significant differences in FC values of left-sided lobule IX and whole brain between the AD and MCI groups (Table 4 and Figure 4).

**Table 4:** Regions showing resting state functional connectivity (rsFC) in cerebellum left lobule IX.

**Brain region (aal)**	**Cluster size**	**MNI coordinates**			***T***
		**X**	**Y**	**Z**	
**ANOVA**					
L-Parietal lobe	116	−3	−48	48	8.93
**AD < Controls**					
R-Parietal lobe/Precuneus	62	0	−57	51	−3.61
**aMCI < Controls**					
R-Cerebellum anterior lobe	56	9	−45	−33	3.79

*MNI, Montreal Neurological Institute; Cluster size > 100 voxels in ANOVA analysis; significance set at p < 0.05 corrected by AlphaSim; Cluster size >30 voxels in two-sample T-test. Significance set at p < 0.05 corrected by Bonferroni*.

#### Functional Connectivity Changes of the Right Lobule IX

**Figure 5:**
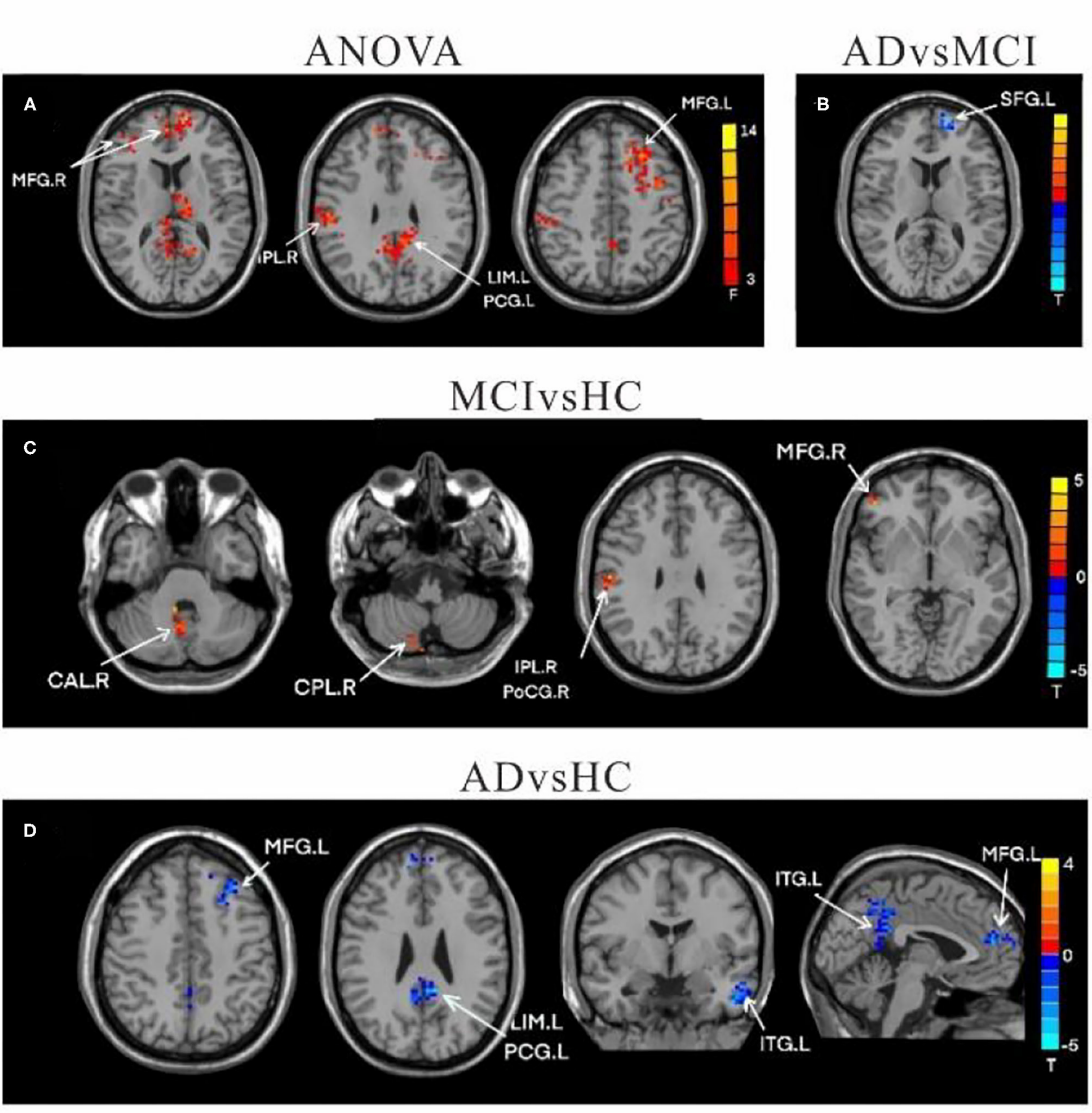
Differences of FC values in the cerebellum right lobule IX. **(A)** Brain regions showing significant differences in FC of the right lobule IX based on analysis of variance (ANOVA) analysis between HC, AD, and aMCI; *p* < 0.05, the cluster size > 100 voxels. **(B–D)** The results of *post-hoc* two-sample *T*-tests in voxel-wise analysis (Bonferroni corrected, cluster size ≥ 30 voxels, *p* < 0.05). AD, Alzheimer's disease; aMCI, amnestic mild cognitive impairment; HC, healthy controls; MFG, Middle Frontal Gyrus; IPL, inferior parietal lobe; LIM, limbic lobe; PCG, posterior cingulate gyrus; SFG, superior frontal gyrus; CAL, cerebellum Anterior lobe; CPL, cerebellum posterior lobe; THA, Thalamus; ITG, inferior temporal gyrus; CAU, Caudate; L, left; R, right.

Compared to HC, the FC between right-sided lobule IX and left inferior temporal gyrus (ITG), left LIM/Posterior Cingulate Gyrus (PCG), left MFG, and right SFG was decreased among all AD patients. However, in comparison to HC, the MCI group had increased FC between the right-sided lobule IX and right parietal lobe/postcentral gyrus, right MFG, right CPL, and right CAL, while there was decreased FC between the right lobule IX and left SFG FC values (Table 5 and Figure 5).

**Table 5:** Regions showing resting state functional connectivity (rsFC) in cerebellum right lobule IX.

**Brain region (aal)**	**Cluster size**	**MNI coordinates**			***T***
		**X**	**Y**	**Z**	
**ANOVA**					
R-Middle frontal gyrus	101	48	42	−3	9.67
L-Limbic lobe/Posterior cingulate	472	−9	−45	27	15.91
R-Medial frontal gyrus	178	3	51	12	10.99
R-Parietal lobe/Postcentral gyrus	107	57	−18	30	10.63
L-Middle frontal gyrus	196	−24	24	48	10.95
**AD < Controls**					
L- Inferior temporal gyrus	53	−48	−3	−36	−3.78
L-Limbic lobe/Posterior cingulate gyrus	169	−9	−45	27	−4.46
L- Medial frontal gyrus	88	−3	48	15	−3.85
L- Middle frontal gyrus	83	−30	30	45	−3.5
R- Superior frontal gyrus	31	21	21	39	−3.54
**aMCI < Controls**					
R-Cerebellum posterior lobe (Crus2)	33	6	−84	−48	3.21
R-Cerebellum anterior lobe	31	9	−45	−33	4.13
R-Middle frontal gyrus	31	48	42	−3	4.13
R-Parietal lobe/Post-central gyrus	53	57	−18	30	4.01
**AD < aMCI**					
L-Superior frontal gyrus	35	−9	63	12	−3.23

*MNI, Montreal Neurological Institute; Cluster size >100 voxels in ANOVA analysis; significance set at p < 0.05 corrected by AlphaSim; Cluster size >30 voxels in two-sample T-test. Significance set at p < 0.05 corrected by Bonferroni*.

#### Correlation Analysis With Clinical Behavior Scores and CSF Biomarkers

**Figure 6:**
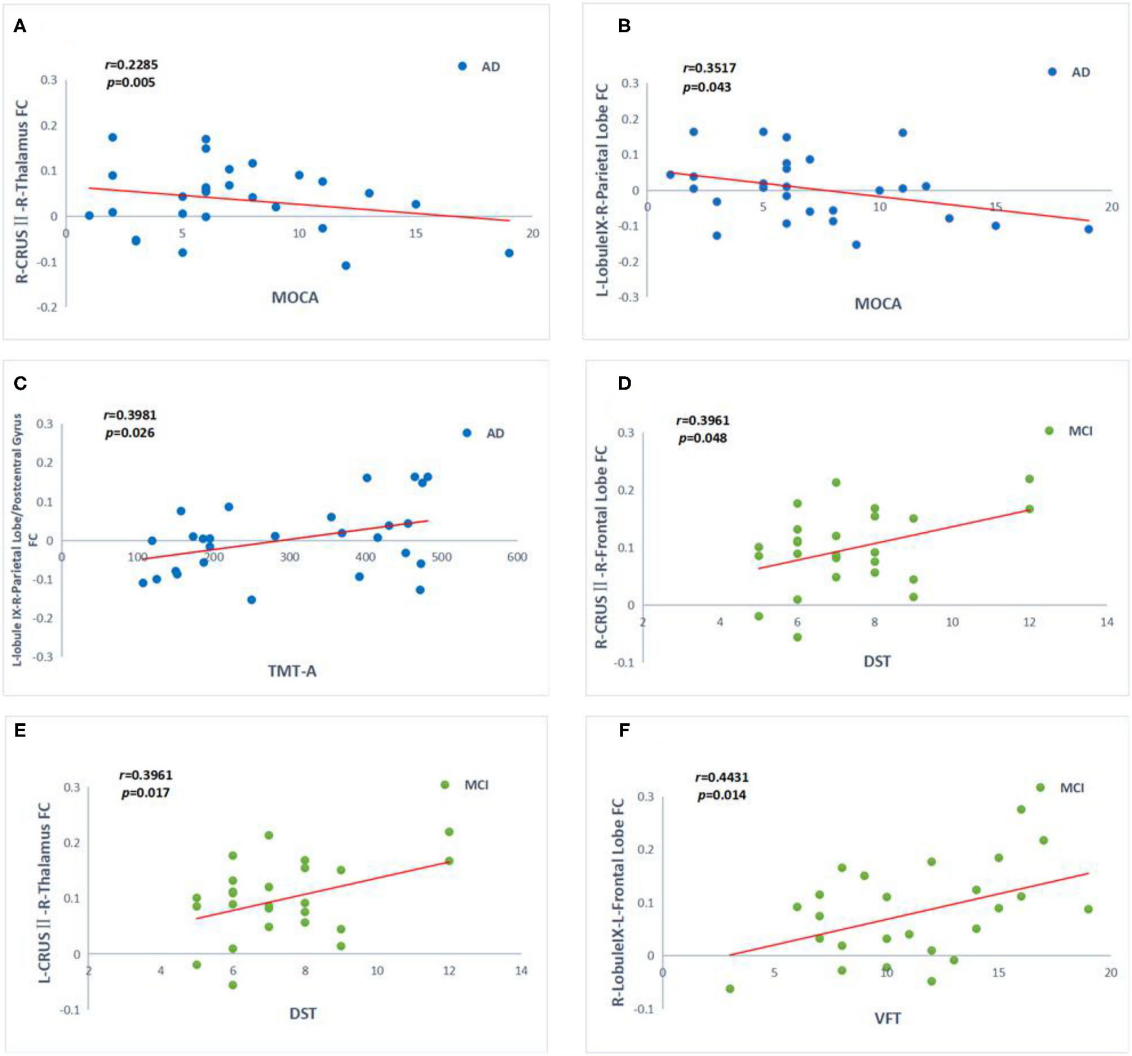
Correlation between the functional connectivity (FC) and cognitive function scores in the AD (blue point) and aMCI (green point) patients. **(A–F)** Significant correlation between FC changes and cognitive function scales include MOCA, STT-A, DST, VFT in cerebellum crus II and lobule IX (Bonferroni corrected, *p* < 0.05). Age, gender, and years of education were used to control variables of the results. AD, Alzheimer's disease; aMCI, amnesic mild cognitive impairment; MOCA, Montreal Cognitive Assessment; DST, Digit Span Test; VTF, Verbal Fluency Test; TMT, Trail Making Test; R, right; L, left.

As shown in Figure 6, MOCA scores negatively correlate with FC values between right crus II and right THAL values (*r* = −0.2285, *p* = 0.005), as well as between left lobule IX and right parietal lobe FC values in AD patients (*r* = −0.3517, *p* = 0.043). In contrast, TMT-A scores positively correlate with FC values between left lobule IX and right parietal lobe/post-central gyrus FC values in AD patients (*r* = 0.3981, *p* = 0.026). In the MCI group, DST scores were positively correlated with FC values between left crus II and right THAL values (*r* = 0.3961, *p* = 0.017), as well as between right crus II and right frontal lobe (*r* = 0.3961, *p* = 0.048). Moreover, VFT scores were also positively correlated with FC values between right lobule IX and left frontal lobe in MCI patients (*r* = 0.4431, *p* = 0.014). There was no significant correlation between CSF biomarkers and functional connectivity (*P* > 0.05).

## Discussion

In the present study, alterations in FC between the cerebellum cognitive sub-region and whole brain were investigated among patients with AD and MCI. We also explored the relevance of this change to cognitive function. Firstly, we found that after adjusting for age, gender, education level and gray matter volume, FC changed brain regions were distributed in temporal region and front-oparietal lobe region, which were important brain regions of DMN and FPN, respectively. Secondly, we identified that FC changes was associated with impaired cognitive function, especially in attention, executive, and memory tasks.

The current research indicates that there are significant changes with regards to the FC of cerebellar cognitive subregions within the AD and MCI groups. Compared to the HC group, FC in frontal lobe, temporal lobe, and parietal lobe were decreased in AD group. Interestingly, the FC between cerebellum and superior frontal gyrus and inferior frontal gyrus in the MCI group was partially weakened, while some connections in the middle frontal gyrus and parietal lobe were strengthened. The research on brain functional connections of AD is now a hot topic, but there are a few studies that have evaluated the effect of the cerebellum on cognitive function connections.

### Functional Connectivity Changes in the AD Patients

In the present study, decreased FC in the AD group were mainly in frontal, temporal, parietal and precuneus cortex, which were closely related to DMN and FPN (38).

It was found in other longitudinal studies that the evolution of AD spectrum is characterized by progressive loss of functional connectivity in the neocortical association area (58, 59). Compared with HC subjects, the hyper connection found in MCI subjects may be a compensation mechanism for the low efficiency of memory network, especially in the temporoparietal region (60, 61). Previous studies have found that the prefrontal and temporoparietal connectivity of MCI patients is stronger than that of normal people (13, 62), With the progression of the disease, the connectivity is weakened in AD (58, 63, 64).

Furthermore, a strong relationship between DMN, lobule IX, and crus II have been reported previously (7, 32, 47, 65). DMN is a brain resting network that is activated when individuals are not engaged in attending to or responding to external stimuli, and is involved in regulating self-reflection and memory processes (66–68). The DMN network, which consists of the PCUN/PCC, medial prefrontal cortex, lateral temporal and parietal cortices and hippocampus, has been proposed to be the most vulnerable brain network in patients with AD (8, 69, 70). Thus, it can be inferred that diminished cerebellar connectivity to the DMN network in AD patients may be one of the causes involved in cognitive impairment. FPN is a network that is involved in attention and working memory function and consists of lateral prefrontal and parietal cortex. It has also been reported that the FPN is remarkably impaired in AD patients, as reported herein (38, 71).

It is important to note that compared to HC, the connections between cognitive subregions of the cerebellum and the limbic lobe were significantly reduced in AD patients. This result is consistent with a recent study that reported that FC in the cerebellar-limbic network was significantly more vulnerable in AD patients compared to aMCI patients (72).

### Functional Connectivity Changes in the MCI Patients

In comparison to HC, patients with MCI show right cerebellar crus II hyper-connectivity with bilateral IPL and MFG, which is functionally associated with FPN. Moreover, the FC between the cerebellum and thalamus, superior frontal gyrus, and inferior frontal gyrus were decreased in MCI patients.

Previous studies have shown that there is less structural and pathological damage at the aMCI stage (5). It has also been reported that increased cerebellar activity was positively correlated with memory enhancement, and served as a compensatory process (58). Thus, we can speculate that an increase in connectivity here may underline some of the compensatory mechanism of the cerebellum for sites of weakened connectivity.

While the FC in IPL sites were impaired in AD patients, they were actually enhanced in aMCI patients, suggesting that enhanced connectivity between the cerebellum and IPL in preclinical AD may have functional compensatory mechanisms (5). The IPL is an important node in DMN and FPN, and is considered a heterogeneous brain area with functions in episodic memory, semantic processing, and spatial cognition (73, 74). Consistent with our results, hyperactivity in the inferior and superior parietal lobes in MCI patients has been reported within the present study (14). Therefore, these findings indicate that IPL may be one of the brain areas responsible for episodic memory. It is worth noting that the aMCI group showed increased FC between the cerebellum and IPL in the DMN, while it was decreased in the AD group, compared to the HC group. This proves that changes in FC may serve as markers for identifying patients with AD and aMCI.

In the present study, decreased FC between the cerebellum and MFG is indicated in AD patients. However, the FC between the right-sided lobule IX and MFG was significantly increased in MCI patients, which may compensate for the impaired memory that is often seen in MCI patients. Previous studies have indicated that decreased DMN connectivity is associated with increased prefrontal connectivity and that this increased connectivity may be a compensatory effect on cognition of the prefrontal lobes (5, 75). In addition, some studies found that FC increased in anterior DMN and FC decreased in posterior DMN; The increase of FC in anterior DMN is considered to be a compensatory increase of cognitive function to maintain task performance (76, 77).

Hypo-connectivity between the cerebellum crus II, lobule IX, and THAL was also investigated in the present study. The cerebellum is involved in cognitive processes through cerebellum-thalamo-cortical pathways to cognitive functions regions, including the prefrontal and parietal cortices, and the cingulate and para-hippocampal gyri (14, 21). The thalamic nuclei are important intermediate stations in the cerebrocerebellar feedback limb of the cerebrocerebellar circuit (33). It is significant for the cerebellum-thalamo-cortical, and cortical-ponto-cerebellar pathways to modulate cognitively-relevant prefrontal and parietal activities (46). Therefore, reduced connectivity between cerebellum and thalamus in AD patients may directly interrupt the connectivity of the cerebellum to cognitive networks, including the DMN and the limbic lobe network, which leads to cognitive impairment.

Further correlation analysis with clinical score revealed that compensation and decompensation in AD and MCI patients were clinically distinguishable. The FC values between the right crus II and the left SFG were positively correlated with DST scores in MCI patients, while the resting-state FC values between the right crus II and the right THAL were negatively correlated with MOCA scores in AD patients. These results indicate that remarkable changes occur in the attentive networks involving lobule VI, crus I, and crus II, implying a fundamental role for these cerebellar areas in attention (78). FC between the cerebellum and fronto-parietal cortex was proposed to be strongly associated with magnitude of cerebellar activation through working memory and attention (79). No correlation with HDAS was found in this study, which may be due to the limited range of the dependent variable in our cohort, which has been added to the revised comments.

We also correlated signals that were extracted from areas of significant FC with cerebrospinal fluid biomarkers, including Aβ1-40, Aβ1-42, Aβ1-40/Aβ1-42, t-tau, and p-tau, which be used as one of the differentiations and diagnostic criteria for Alzheimer's disease (80). However, no statistically significant difference was identified.

Di Lorenzo et al. used the neurophysiological method of short latency afferent inhibition (SAI) and found that stimulating the cerebellum θ Wave group (TBS) can activate cerebello thalamic cortical pathway and regulate central cholinergic function (81). In subsequent study, they used repetitive and paired pulse transcranial magnetic stimulation in patients with different degrees of AD and were followed up for 3 years. The results showed that the loss of LTP like cortical plasticity in AD patients was more severe than that in MCI patients (82). After the study of cerebellum iTBS, they found that the plasticity mechanism of cerebellum cortex was damaged in Alzheimer disease (83). A previous study that applied continuous theta burst stimulation (cTBS) (a non-invasive stimulus in which cTBS can inhibits brain excitability) to the lateral part of the cerebellum found that FC between the frontal and parietal cognitive areas was significantly attenuated, while FC between cTBS and motor areas remained unchanged (84). This also proves that cerebellar TBS can promote the reorganization of cerebellar cortex and has a potential role in improving motor and learning (85). The results of this study demonstrate the role of the cerebellum in AD progression and pathogenesis, and provide a novel target for non-pharmacological interventions. Our findings are able to help explain the mechanism by which non-invasive stimulation improves cognitive impairment in AD, and provides new targets and ideas for non-pharmacological interventions.

## Limitations

There were three major limitations of this study that need to be addressed in the future. Firstly, some patients with ad also have emotional symptoms. The relationship between cerebellum and emotion has been confirmed. Therefore, in the next study, we will add CCAS scale to evaluate more comprehensively. Secondly, in this study, the result of cluster sizes was relatively small, it may be related to the small sample size. We will continue to increase the sample size in the follow-up study. Thirdly, the study was a cross-sectional study, longitudinal studies are needed in the future to study the dynamic changes of cerebellum in the development of AD.

## Conclusion

In conclusion, FC between the cerebellar and other cognitively relevant sub-regions was found to be significantly reduced in AD patients. In MCI patients, the FC between the cerebellum and cortex, including the superior and inferior frontal gyri, was also disrupted. On the other hand, the FC between the cerebellum and the middle frontal gyrus, as well as the parietal regions, was enhanced. These results suggest that MCI may be in the preclinical stage of structural damage and functional compensation of AD, and is associated with cognitive function. And the FC changes in specific cerebello-cortical functional modules may play an important role in the pathogenesis of AD.

## Data Availability Statement

The raw data supporting the conclusions of this article will be made available by the authors, without undue reservation.

## Ethics Statement

The studies involving human participants were reviewed and approved by Medical Research Ethical Committee of Nanjing Brain Hospital in Nanjing, China. The patients/participants provided their written informed consent to participate in this study.

## Author Contributions

JS, FT, and QY designed this study. FT, WM, and QL analyzed the data. FT and DZ wrote the manuscript. All authors contributed to the article and approved the submitted version.

## Conflict of Interest

The authors declare that the research was conducted in the absence of any commercial or financial relationships that could be construed as a potential conflict of interest.
